# Supplementary material for: Clinical features associated with NeoRAS wild-type metastatic colorectal cancer A SCRUM-Japan GOZILA substudy
Source: Nat Commun. 2024 Jul 13;15:5885. doi: 10.1038/s41467-024-50026-4 (PMC11246505; doi:10.1038/s41467-024-50026-4)
Supplement: Supplementary file 1 — Supplementary Information [file 41467_2024_50026_MOESM1_ESM.pdf]

Supplementary Information

Supplementary Table 1
Description: Presence of gene alterations related to the colorectal cancer in Case 1

Supplementary Table 2
Description: Presence of gene alterations related to the colorectal cancer in Case 2

Supplementary Table 3
Description: Summary of previous studies on Neo*RAS* WT metastatic colorectal cancer

Supplementary Notes
Description: The procedure of whole-exome sequencing

Supplementary Table 1. Presence of gene alterations related to the colorectal cancer in Case 1

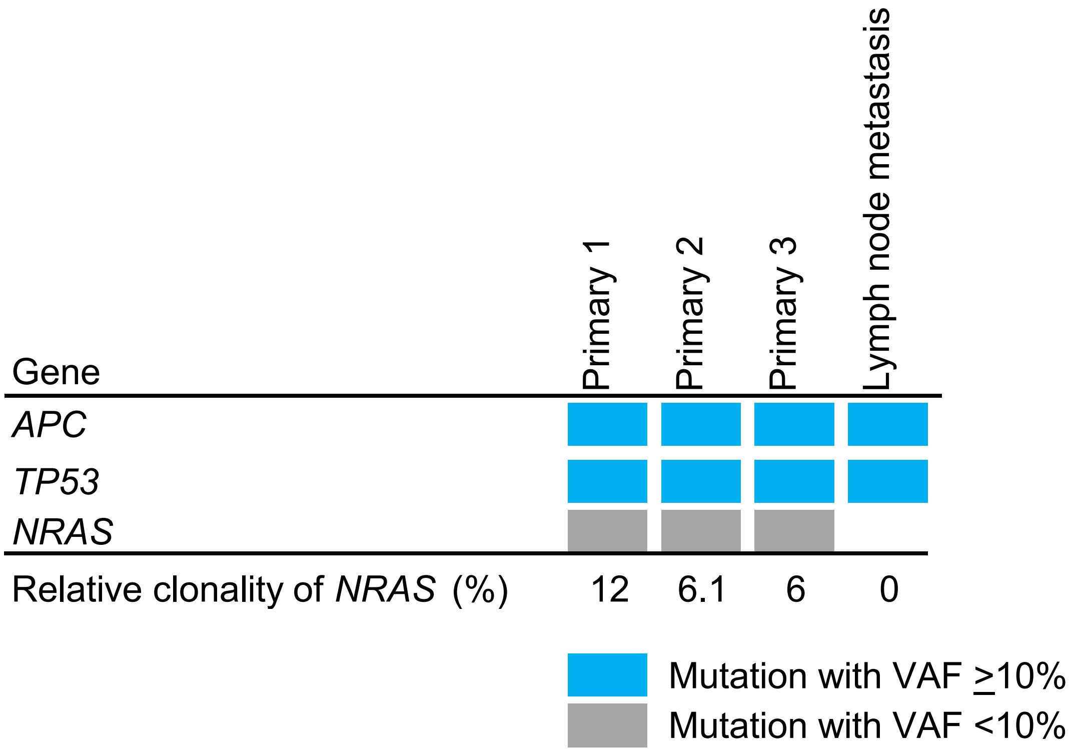

Supplementary Table 2. Prescence of gene alterations related to the colorectal cancer in Case 2

Gene	Primary 1	Primary 2	Primary 3	Lymph node metastasis	Ovarian metastasis 1	Ovarian metastasis 2	Liver metastasis 1	Liver metastasis 2	Lung metastasis 1	Lung metastasis 2
NRAS	■	■	■	■	■	■	□		■	■
TTN	■	■	■	■	■	■	□		■	■
TP53	■	■	■	■	■	■			■	■
APC	■	■	■	□	■	■			■	■
POLE	■		■			■	□			
ATM		■	■			■				
MAP7	□	■	■						□	
ARID1A	■	□				■				
MYO1B	■	□				□				
TCERG1	■			□					□	
MSH4	■		■							
MSH6	■		■							
PIK3CA					■	■				
PTPN12		■				■				
ACVR2A		□			■					
CTNNB1					■	□				
FZD3					■			□		
MLH1			■		□					
SMAD4	■	□								
CASP8						■				
CDC27		■								
KIAA1804						■				
MIER3						■				
MLH3		■								
MSH2			■							
MSH3						■				
MSH5			■							
TGFBR2			■							
Relative clonality of NRAS (%)	88	100	60	59	47	72	27	0	76	75

■ Mutation with VAF ≥10%
□ Mutation with VAF <10%

Supplementary Table 3. Summary of previous studies on Neo*RAS* WT metastatic colorectal cancer

References	Number of patients	ctDNA assay	Neo*RAS* WT (%), *RAS* only	Neo*RAS* WT (%), *RAS* and ctDNA presence	Method to confirm ctDNA presence
Fernández et al.	16	BEAMing	3 (18.8)	N/A	N/A
Sunakwa et al.	29	Real-time PCR	18 (62.1)	N/A	N/A
Sato et al.	64	BEAMing	27 (43.5)	N/A	N/A
Osumi et al.	107	BEAMing	23 (21.5)	N/A	N/A
Raimondi et al.	11	Real-time PCR	5 (45.5)	1 (9.1)	Other somatic alterations
Nicolazzo et al.	12	Real-time PCR	10 (83.3)	5 (41.7)	Methylated genes
Moati et al.	36	NGS or ddPCR	8 (22.2)	2 (5.6)	Other somatic alterations or methylated genes
Jason et al.	74	NGS	23 (31.1)	1 (1.4)	Other somatic alterations

Abbreviations, *RAS*: rat sarcoma viral oncogene homolog, WT: wild-type, PCR: polymerase chain reaction, BEAMing: beads, emulsion, amplification, and magnetics, NGS: next-generation sequencing, ctDNA: circulating tumor DNA, NA: not available

Supplementary Notes.

Whole-exome sequencing was performed as previously described. Genomic DNA was extracted from the patients' tumor cells obtained from the primary surgical tissue and metastatic site (lymph node, liver, lung, and ovary) using the AllPrep DNA/RNA FFPE Kit (QIAGEN). Normal control genomic DNA was extracted from the patients' normal tissues. Whole-exome libraries were prepared from genomic DNA using the SureSelect Human All Exon V6 kit (Agilent Technologies) according to the manufacturer's instructions. The prepared whole-exome libraries were sequenced using 150-bp paired-end reads on a NextSeq or NovaSeq sequencer (Illumina). For whole-exome sequencing, sequence reads were mapped to the human reference genome GRCh37/hg19 using Burrows–Wheeler Aligner (v0.7.10). Possible PCR duplicates, read pairs with a mapping quality of <30, and mismatches of >5% were excluded. Mutation calling was performed as described previously using the following parameters: (i) base quality ≥ 15; (ii) sequence depth ≥10; (iii) variant depth ≥ 4; (iv) variant frequency in a tumor ≥ 10%; (v) variant frequency in normal samples < 2%; and (vi) Fisher's *P*-value < 0.05. Indels and splicing region were annotated using ANNOVAR. Somatic mutations were classified as follows: (1) non-synonymous single-nucleotide variants, (2) splicing, (3) stop gain, (4) stop loss, (5) insertion/deletion, and (6) synonymous single-nucleotide variants. A splicing mutation indicated a single-nucleotide variation at the position of the splicing acceptor of the donor site.
