# Supplementary material for: Clinical features associated with NeoRAS wild-type metastatic colorectal cancer A SCRUM-Japan GOZILA substudy
Source: Nat Commun. 2024 Jul 13;15:5885. doi: 10.1038/s41467-024-50026-4 (PMC11246505; doi:10.1038/s41467-024-50026-4)
Supplement: Supplementary file 3 — Reporting Summary [file 41467_2024_50026_MOESM3_ESM.pdf]

nature portfolio

Corresponding author(s): Eiji Shinozaki, Takayuki Yoshino

Last updated by author(s): 11. Mar. 2024

Reporting Summary

Nature Portfolio wishes to improve the reproducibility of the work that we publish. This form provides structure for consistency and transparency in reporting. For further information on Nature Portfolio policies, see our Editorial Policies and the Editorial Policy Checklist.

Please do not complete any field with "not applicable" or n/a. Refer to the help text for what text to use if an item is not relevant to your study.
For final submission: please carefully check your responses for accuracy; you will not be able to make changes later.

Statistics

For all statistical analyses, confirm that the following items are present in the figure legend, table legend, main text, or Methods section.

n/a	Confirmed	
☐	☒	The exact sample size (*n*) for each experimental group/condition, given as a discrete number and unit of measurement
☒	☐	A statement on whether measurements were taken from distinct samples or whether the same sample was measured repeatedly
☐	☒	The statistical test(s) used AND whether they are one- or two-sided<br>*Only common tests should be described solely by name; describe more complex techniques in the Methods section.*
☐	☒	A description of all covariates tested
☒	☐	A description of any assumptions or corrections, such as tests of normality and adjustment for multiple comparisons
☐	☒	A full description of the statistical parameters including central tendency (e.g. means) or other basic estimates (e.g. regression coefficient) AND variation (e.g. standard deviation) or associated estimates of uncertainty (e.g. confidence intervals)
☐	☒	For null hypothesis testing, the test statistic (e.g. *F*, *t*, *r*) with confidence intervals, effect sizes, degrees of freedom and *P* value noted<br>*Give P values as exact values whenever suitable.*
☒	☐	For Bayesian analysis, information on the choice of priors and Markov chain Monte Carlo settings
☐	☒	For hierarchical and complex designs, identification of the appropriate level for tests and full reporting of outcomes
☒	☐	Estimates of effect sizes (e.g. Cohen's *d*, Pearson's *r*), indicating how they were calculated

Our web collection on statistics for biologists contains articles on many of the points above.

Software and code

Policy information about availability of computer code

Data collection	Ion ReporterTM Software version 4.4 (for OCA v1) and 5.0 (for OCA v3), Guardant360 (version 2.10 and 2.11), RTA software version 2.12, bcl2fastq version 2.19., BWA-MEM version 0.7.15 (arXiv:1303.3997v2). Clinical data were collected using Viedoc version 4.57 for GI-SCREEN and Trial Master version 5.0 for GOZILA.
Data analysis	Statistical software “EZR” (Saitama Medical Center, Jichi Medical University, Saitama, Japan) in R and R commander.

For manuscripts utilizing custom algorithms or software that are central to the research but not yet described in published literature, software must be made available to editors and reviewers. We strongly encourage code deposition in a community repository (e.g. GitHub). See the Nature Portfolio guidelines for submitting code & software for further information.

Data

Policy information about availability of data

All manuscripts must include a data availability statement. This statement should provide the following information, where applicable:

- Accession codes, unique identifiers, or web links for publicly available datasets
- A description of any restrictions on data availability
- For clinical datasets or third party data, please ensure that the statement adheres to our policy

The authors declare that all data supporting the findings of this study are available within the article and its supplementary information. To protect the privacy and confidentiality of patients in this study, clinical data was not made publicly available. in a repository or are provided in the supplementary material of the article.

Research involving human participants, their data, or biological material

Policy information about studies with human participants or human data. See also policy information about sex, gender (identity/presentation), and sexual orientation and race, ethnicity and racism.

Reporting on sex and gender.	Our study is a clinical study with human participants who self-reported their biological sex on the requisition form upon enrollment. Information on the breakdown of biological sex in our cohort is detailed in Table 1 where 52.1% (249/478) of the cohort were male and the remainder were female.
Population characteristics.	Population characteristics are provided in Table 1.
Recruitment	All registration was done on a voluntary basis by researchers at each participating site. The possibility of bias in the selection by researchers cannot be ruled out. However, since enrollment was prospective and each site enrolled most of the cases that fit the criteria, we believe that bias was suppressed as much as possible.
Ethics oversight	This study was approved by the Institutional Review Board of the Cancer Institute Hospital of the Japanese Foundation of Cancer Research (IRB number: 2021-GB-009) and was conducted in accordance with the principles of the Declaration of Helsinki. The protocol was described on the hospital's website, and as the participants were given the opportunity to opt out of the study, informed consent was not obtained in this study.

Note that full information on the approval of the study protocol must also be provided in the manuscript.

Field-specific reporting

Please select the one below that is the best fit for your research. If you are not sure, read the appropriate sections before making your selection.

☒ Life sciences ☐ Behavioural & social sciences ☐ Ecological, evolutionary & environmental sciences

For a reference copy of the document with all sections, see nature.com/documents/nr-reporting-summary-flat.pdf

Life sciences study design

All studies must disclose on these points even when the disclosure is negative.

Sample size.	As the study was observed, a sample size calculation based on the formal statistical testing was not performed. Among the 4991. patients with mCRC enrolled in GOZILA between March 2018 and February 2022, 647 were initially diagnosed with *RAS* MT mCRC using pretreatment tissue analysis. Among them, 169 patients in whom ctDNA was measured before the administration of first-line chemotherapy (*N*=152) and in whom plasma *RAS* MTs were different from the tissue *RAS* MTs (*N*=17) were excluded. Finally, 478 patients were evaluated. Largest number of cases amongst previous reports examining NeoRAS WT mCRC, and the number of cases was judged to be sufficient for the needs of the study.
Data exclusions.	Patients in whom ctDNA was measured before the administration of first-line chemotherapy and in whom plasma *RAS* MT were different from the tissue *RAS* MT were excluded.
Replication.	This study was a clinical study with human participants. Hence, replication of data set is not applicable for our study. No residual blood samples remained, making re-examination difficult.
Randomization	This is an observational study and is not a randomized trial.
Blinding.	As described above, we believe randomization was essentially difficult in this study. Accordingly, no blinding was used.

Reporting for specific materials, systems and methods

We require information from authors about some types of materials, experimental systems and methods used in many studies. Here, indicate whether each material, system or method listed is relevant to your study. If you are not sure if a list item applies to your research, read the appropriate section before selecting a response.

n/a	Involved in the study	
☐	☒	Antibodies
☒	☐	Eukaryotic cell lines
☒	☐	Palaeontology and archaeology
☒	☐	Animals and other organisms
☐	☒	Clinical data
☒	☐	Dual use research of concern
☒	☐	Plants

n/a	Involved in the study	
☒	☐	ChIP-seq
☒	☐	Flow cytometry
☒	☐	MRI-based neuroimaging

Clinical data

Policy information about clinical studies

All manuscripts should comply with the ICMJE guidelines for publication of clinical research and a completed CONSORT checklist must be included with all submissions.

Clinical trial registration. UMIN000029315 for GOZILA

Study protocol. The protocol was described on the hospital's website.

Data collection. Clinical and genomic data were collected from 4,991 patients in GOZILA between March 2018 and February 2022.

Outcomes. Neo*RAS* WT is prevalent in 9.8% of mCRC patients with sufficient tumor DNA shedding. The absence of liver metastasis and tissue *RAS* MTs are associated with the emergence of Neo*RAS* WT. Anti-EGFR mAb-based therapy may be effective in Neo*RAS* WT mCRC.

This checklist template is licensed under a Creative Commons Attribution 4.0 International License, which permits use, sharing, adaptation, distribution and reproduction in any medium or format, as long as you give appropriate credit to the original author(s) and the source, provide a link to the Creative Commons license, and indicate if changes were made. The images or other third party material in this article are included in the article's Creative Commons license, unless indicated otherwise in a credit line to the material. If material is not included in the article's Creative Commons license and your intended use is not permitted by statutory regulation or exceeds the permitted use, you will need to obtain permission directly from the copyright holder. To view a copy of this license, visit http://creativecommons.org/licenses/by/4.0/
