# Supplementary material for: Clinical features associated with NeoRAS wild-type metastatic colorectal cancer A SCRUM-Japan GOZILA substudy
Source: Nat Commun. 2024 Jul 13;15:5885. doi: 10.1038/s41467-024-50026-4 (PMC11246505; doi:10.1038/s41467-024-50026-4)
Supplement: Supplementary file 4 — Description of Additional Supplementary Files [file 41467_2024_50026_MOESM4_ESM.pdf]

Description of Additional Supplementary Files

File Name: Supplementary Data 1

Description: Comparison about clinicodemographic patient characteristics between Neo*RAS* wild type and *RAS* mutant metastatic colorectal cancer in Group A and B.

File Name: Supplementary Data 2

Description: Comparison about clinicodemographic patient characteristics between *RAS* wild type, Neo*RAS* wild type and *RAS* mutant metastatic colorectal cancer in Group A and B.

File Name: Supplementary Data 3

Description: Comparison between tissue and ctDNA genomic data.
